# Generation of a novel human dermal substitute functionalized with antibiotic-loaded nanostructured lipid carriers (NLCs) with antimicrobial properties for tissue engineering

**DOI:** 10.1186/s12951-020-00732-0

**Published:** 2020-11-23

**Authors:** Jesús Chato-Astrain, Isabel Chato-Astrain, David Sánchez-Porras, Óscar-Darío García-García, Fabiola Bermejo-Casares, Claudia Vairo, María Villar-Vidal, Garazi Gainza, Silvia Villullas, Roke-Iñaki Oruezabal, Ángela Ponce-Polo, Ingrid Garzón, Víctor Carriel, Fernando Campos, Miguel Alaminos

**Affiliations:** 1 Tissue Engineering Group, Department of Histology, Faculty of Medicine, University of Granada, Avenida de la Investigación 11, 18016 Granada, Spain; 2 Instituto de Investigación Biosanitaria Ibs.GRANADA, Granada, Spain; 3 BioKeralty Research Institute AIE, Albert Einstein, 25-E3, 01510 Miñano, Spain; 4 Keralty Health SI, Barrio Arkauti, 5, 01192 Vitoria-Gasteiz, Spain; 5 Red Andaluza de Diseño Y Traslación de Terapias Avanzadas, Sevilla, Spain

**Keywords:** Tissue engineering, Functionalization, Dermal substitute, Severe burns, Human skin, Nanostructured lipid carriers, Colistimethate, Amikacin

## Abstract

**Background:**

Treatment of patients affected by severe burns is challenging, especially due to the high risk of *Pseudomonas* infection. In the present work, we have generated a novel model of bioartificial human dermis substitute by tissue engineering to treat infected wounds using fibrin-agarose biomaterials functionalized with nanostructured lipid carriers (NLCs) loaded with two anti-*Pseudomonas* antibiotics: sodium colistimethate (SCM) and amikacin (AMK).

**Results:**

Results show that the novel tissue-like substitutes have strong antibacterial effect on *Pseudomonas* cultures, directly proportional to the NLC concentration. Free DNA quantification, WST-1 and Caspase 7 immunohistochemical assays in the functionalized dermis substitute demonstrated that neither cell viability nor cell proliferation were affected by functionalization in most study groups. Furthermore, immunohistochemistry for PCNA and KI67 and histochemistry for collagen and proteoglycans revealed that cells proliferated and were metabolically active in the functionalized tissue with no differences with controls. When functionalized tissues were biomechanically characterized, we found that NLCs were able to improve some of the major biomechanical properties of these artificial tissues, although this strongly depended on the type and concentration of NLCs.

**Conclusions:**

These results suggest that functionalization of fibrin-agarose human dermal substitutes with antibiotic-loaded NLCs is able to improve the antibacterial and biomechanical properties of these substitutes with no detectable side effects. This opens the door to future clinical use of functionalized tissues. 
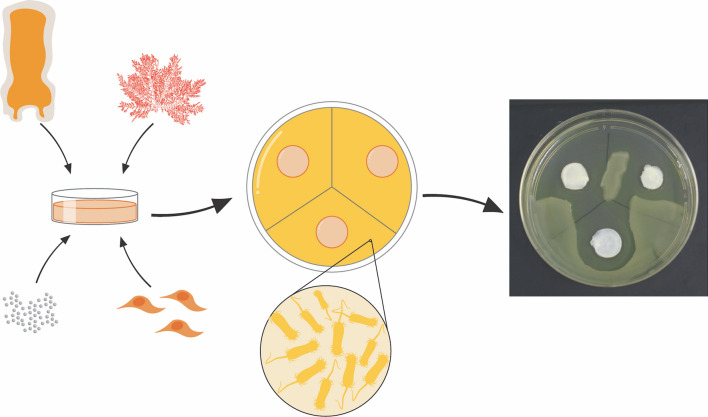

## Background

Tissue engineering strategies attempt to generate biologically active tissue-like substitutes to restore and maintain the normal function of damaged tissues and organs [1]. Tissue engineering protocols combine the principles of engineering and biological sciences to create artificial substitutes based on three main components: cells, biocompatible scaffolds and bioactive factors [1–4].

Among the different biomaterials that showed preclinical and clinical usefulness for the generation of human tissue substitutes, fibrin-agarose hydrogels show excellent biocompatibility and biomechanical properties and its porous fibrillar pattern allows diffusion and interchange of oxygen and nutrients [2, 3, 5, 6]. Fibrin-agarose was previously used to generate bioartificial substitutes of the human cornea [7], sclera [8], oral mucosa [9], palate [10], nerve [11, 12], cartilage [13] and skin [14, 15]. In fact, a bioartificial model of tissue-engineered human skin (UGRSKIN), consisting of a dermal skin substitute based on fibrin-agarose biomaterials and dermal fibroblasts and an overlying epithelial layer, has been successfully translated to the clinical setting [16]. The UGRSKIN model was approved by the National Medicines Agency in Spain (*Agencia Española de Medicamentos y Productos Sanitarios*—AEMPS) and is currently being used as an Advanced Therapy Medicinal Product (ATMP) in patients with severe skin burns [16]. Although preliminary clinical results of this model are very positive, further research is needed in order to enhance the biological properties of these bioartificial tissues and improve their clinical efficiency.

In this regard, one of the main clinical challenges in patients affected by severe skin burns is preventing bacterial infection [17–19]. Specifically, *Pseudomonas aeruginosa* is one of the main pathogens that first colonizes burn wounds and can produce severe respiratory system and systemic complications that can compromise patient's life [17, 18, 20]. However, the treatment of these infections through conventional antibiotic therapies is often difficult due to the wide range of antimicrobial multidrug resistance of this pathogen and the low bioavailability of certain antibiotics [21, 22].

As an alternative against multidrug-resistant bacteria, new drug delivery systems have emerged. Among them, nanostructured lipid carriers (NLCs) have gained attention as promising second-generation of lipid-based delivery systems, allowing efficient drug delivery to target tissues. In particular, NLCs are capable of exerting a local effect when topically administered on a wound, as demonstrated by Vairo et al. [23]. Moreover, antimicrobial activity can be increased when anti-*Pseudomonas* antibiotics are loaded in these nanoparticles by enhancing the interaction between the biofilm-bearing colony and the antibiotics. In addition, nanoencapsulation provides sustained controlled release able to reduce drug dose and thus, decrease drug toxicity and improve the safety profile of well-known antibiotics such as sodium colistimethate (SCM) and amikacin (AMK) [24–27].

In the present work, we have functionalized the previously available fibrin-agarose biomaterial with antibiotic-loaded NLCs in order to provide antimicrobial properties to the biomaterial. Then, the resulting functionalized biomaterial was used to generate a functionalized model of the human skin dermis (FSS) produced by tissue engineering. This novel dermal skin substitute could contribute to improve the clinical performance and usefulness of currently-available fibrin-agarose skin models (SS).

## Results

### Antibiotic-loaded nanoparticle characterization

Nanoparticle size was 132.9 ± 3.4, 123.12 ± 4.11 and 116.8 ± 9.2 nm, for AMK-NLCs, SCM-NLCs and CTR-NLCs, respectively. Dispersion analysis resulted in a satisfactory particle homogeneity for all samples (SPAN < 0.8). Besides, the used preparation method led to a final antibiotic loading of 0.06 mg drug/mg and an encapsulation efficiency of 93.24 ± 2.21 and 77.25 ± 7.04%, for AMK-NLCs and SCM-NLCs, respectively.

### Biocompatibility analysis

Quantification of DNA released to the culture medium as a result of cell damage revealed that most experimental conditions were associated to very low levels of DNA (Table 1). After 24 h, we found that the lowest concentrations of each type of NLCs (10 and 100 µg/ml) were not associated to significant levels of released DNA (differences with positive controls were non-significant, whereas differences with negative controls were statistically significant). However, FSS-AMK1000, FSS-SCM300 and FSS-SCM1000 showed low amounts of DNA released to the medium, although differences with positive controls were statistically significant. At 48 h, we found that the highest concentrations of the three types of NLCs (1000 µg/ml), along with FSS-SCM10 showed low amounts of released DNA, showing statistically significant differences as compared to positive controls. In all cases, the amount of DNA was significantly lower than negative controls.

**Table 1:** Percentage of cytotoxicity found in each group and positive (CTR +) and negative (CTR−) controls as determined by quantification of DNA released to the culture medium (DNA) and WST1 metabolic function after 24 h and 48 h of ex vivo follow-up

	FSS-AMK 10	FSS-AMK 100	FSS-AMK300	FSS-AMK 1000	FSS-SCM 10	FSS-SCM 100	FSS-SCM300	FSS-SCM 1000	FSS-CTR 10	FSS-CTR 100	FSS-CTR300	FSS-CTR 1000	CTR +	CTR-
DNA														
24 h	0 ± 0^a^	0 ± 0^a^	0 ± 0^a^	3.7 ± 0.3^a,b^	0 ± 0^a^	0 ± 0^a^	1.1 ± 0.1^a,b^	0.7 ± 0.2^a,b^	0 ± 0^a^	0 ± 0^a^	0 ± 0^a^	0 ± 0^a^	0 ± 0.2^a^	100 ± 0.5^b^
48 h	0 ± 0^a^	0 ± 0^a^	0 ± 0^a^	4.7 ± 1^a,b^	8.7 ± 1.1^a,b^	0 ± 0^a^	0 ± 0^a^	6 ± 0.8^a,b^	0 ± 0.1^a^	0 ± 0^a^	0 ± 0^a^	1 ± 2.3^a,b^	0 ± 0.1^a^	100 ± 9.2^b^
WST1														
24 h	14.6 ± 7.1^a,b^	8.4 ± 3.7^a,b^	3.7 ± 5.5^a,b^	0 ± 0^a^	8.7 ± 8.2^a,b^	5.8 ± 5.3^a,b^	0 ± 0^a^	0 ± 0^a^	4.6 ± 3.8^a,b^	10.4 ± 9.2^a,b^	6.1 ± 3^a,b^	3.6 ± 3.9^a,b^	0 ± 5.6^a^	100 ± 0.1^b^
48 h	11.1 ± 10.7^a^	0.2 ± 0.6^a^	9.2 ± 3.6^a^	3 ± 3.7^a^	5.9 ± 5.7^a^	2.5 ± 2.6^a^	0 ± 0^a^	0 ± 0^a^	3.4 ± 4.4^a^	7.7 ± 7.3^a^	1.4 ± 2.7^a^	0 ± 0^a^	0 ± 19.1^a^	100 ± 0.1^b^

^a^Differences with negative controls are statistically significant

^b^Differences with positive controls are statistically significant. FSS: functionalized human dermal skin substitutes containing different types of NLCs (AMK, SCM or CTR) at different concentrations (10, 100, 300 or 1000 µg/ml)

For the WST-1 metabolic assay (Table 1), results differed for the two follow-up times evaluated. After 24 h, the metabolic function was partially diminished (less than 15%) in cells in contact with the lowest concentrations (10 and 100 µg/ml) of the three types of NLCs, and for FSS-AMK300, FSS-CTR300 and FSS-CTR1000, as compared to positive controls, with all cases showing significant differences with negative controls. Following 48 h, FSS showed non-significant differences with positive controls, but were significantly lower than negative controls.

### Histological analysis

**Fig. 1:**
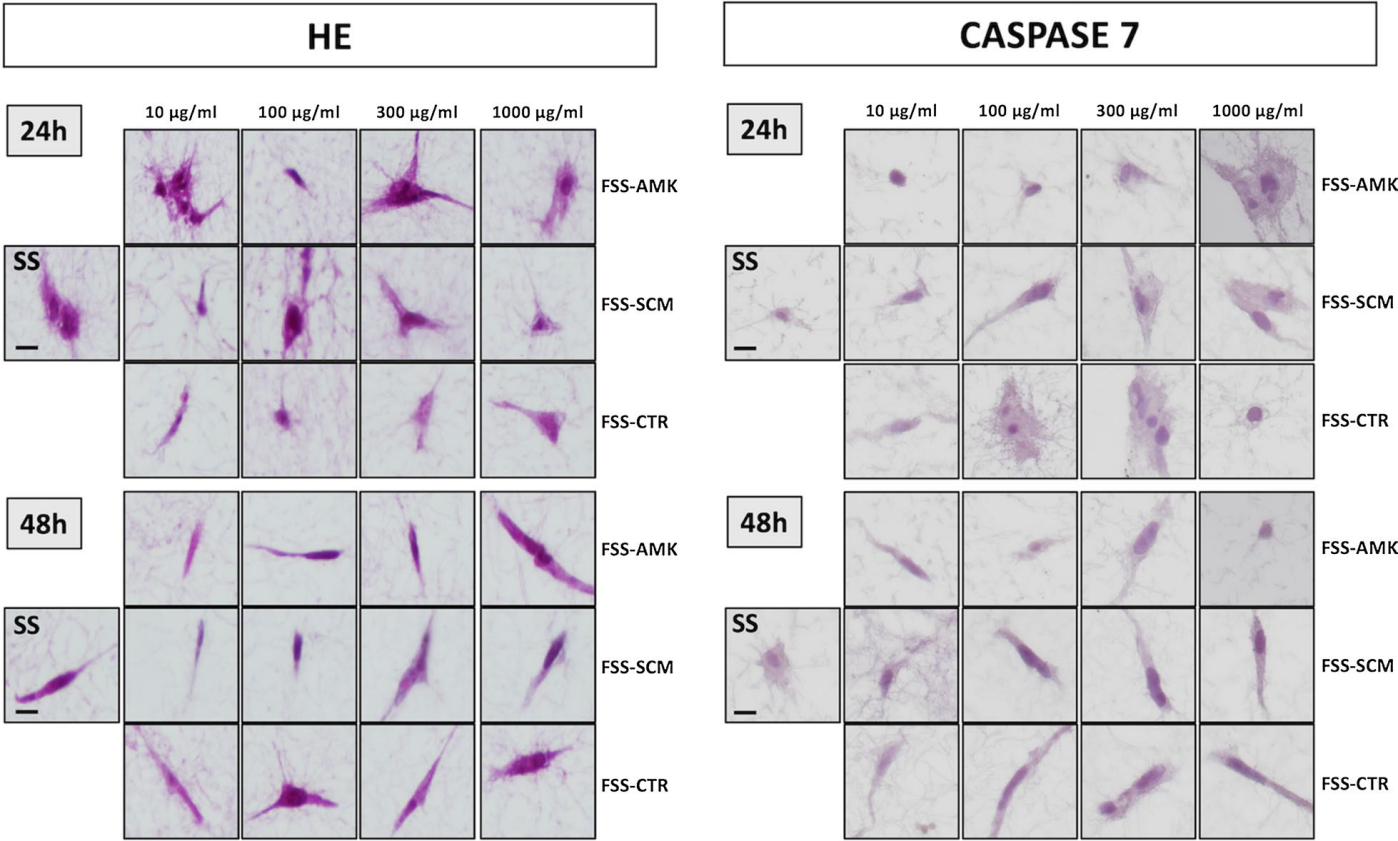
Hematoxylin–eosin histological analysis and caspase 7 immunohistochemistry of dermal fibroblast cells in the different groups. SS: non-functionalized human dermal skin substitutes, FSS: functionalized human dermal skin substitutes containing different types of NLCs (AMK, SCM or CTR) at different concentrations (10, 100, 300 or 1000 µg/ml). Scale bar: 10 µm

Histological analysis of the different bioengineered human dermal skin substitutes generated in the present work revealed that human skin fibroblasts displayed a normal morphology and structure in all experimental conditions (Fig. 1). At 24 h, cells showed the typical elongated or spindle-shape morphology of human fibroblasts, with no differences between FSS and control non-functionalized SS, as occurred after 48 h. In SS and all FSS groups, the fibrin-agarose biomaterial consisted of a fibrillar mesh with abundant porous spaces among the randomly distributed fibrils. Cells were able to spread within the fibrin-agarose biomaterial mesh and cell prolongations (pseudopods or filopodia) were emitted in all spatial directions. No signs of cell death were found in any of the samples. These findings are in agreement with the immunohistochemical analysis of caspase 7 showing negative expression of this apoptosis marker in control non-functionalized skin substitutes and in all FSS groups at 24 and 48 h (Fig. 1).

**Fig. 2:**
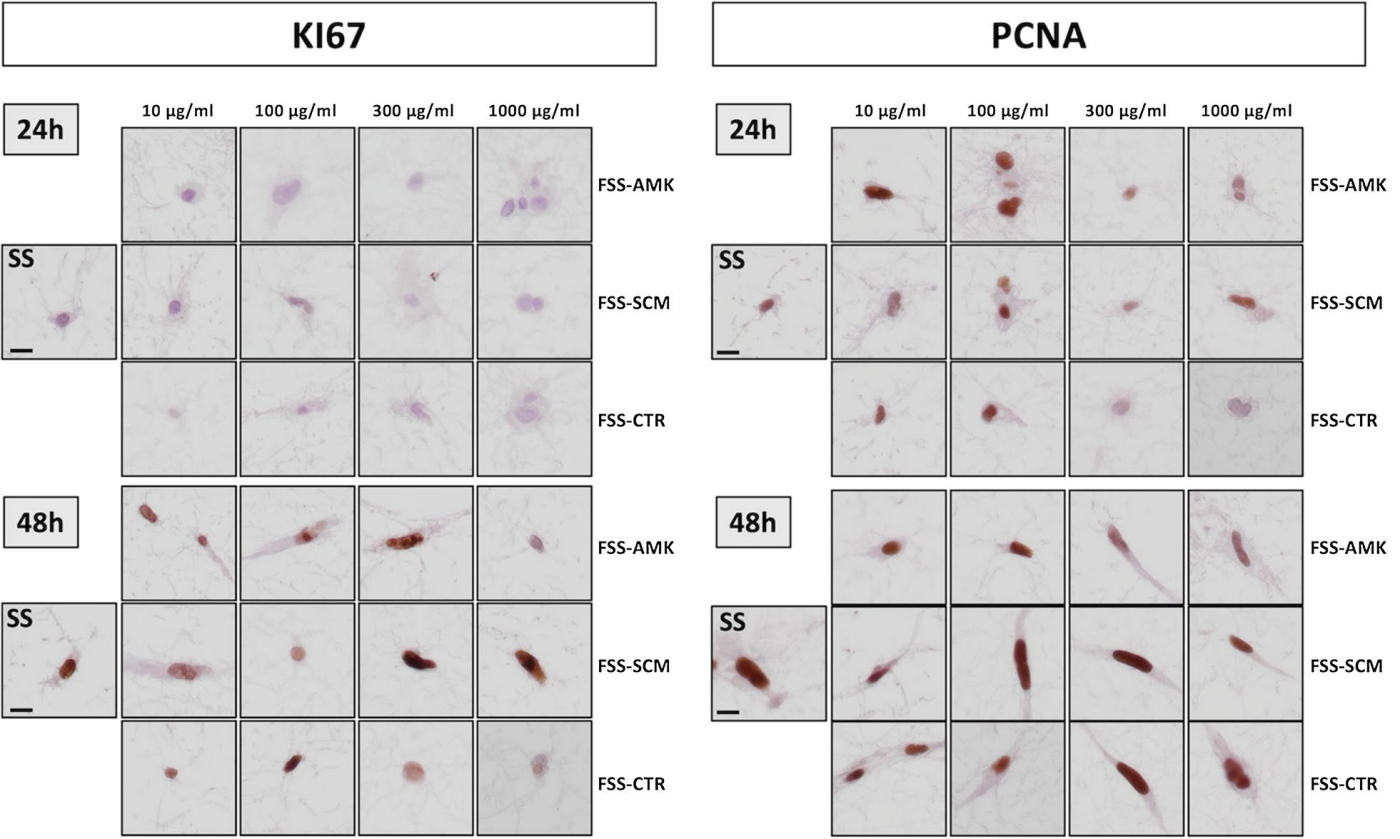
Immunohistochemical analysis of dermal fibroblast cells in the different groups for the cell proliferation markers PCNA and KI67. SS: non-functionalized human dermal skin substitutes, FSS: functionalized human dermal skin substitutes containing different types of NLC (AMK, SCM or CTR) at different concentrations (10, 100, 300 or 1000 µg/ml). Scale bar: 10 µm

On the other hand, cell proliferation was analyzed in control and FSS groups to determine the effect of functionalization on this parameter. In this regard, immunohistochemical analysis of the cell proliferation markers PCNA and KI67 demonstrated that cells were able to proliferate inside all types of tissue substitutes, especially after 48 h. In fact, we found PCNA-positive cells in all study groups at 24 and 48 h, and KI67-positive cells in all groups at 48 h (Fig. 2).

**Fig. 3:**
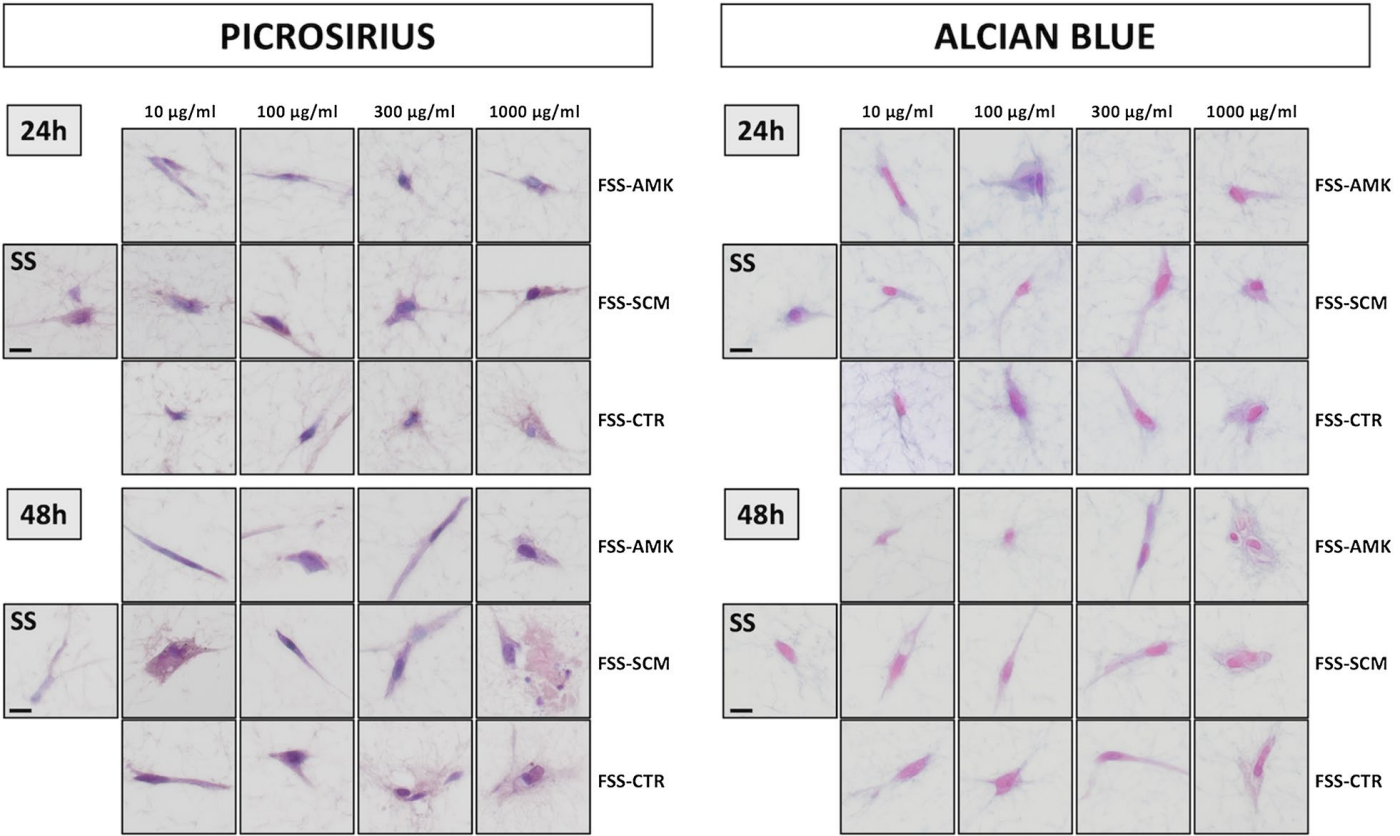
Histochemical analysis of dermal fibroblast cells in the different groups using picrosirius red (for collagen identification) and alcian blue (for proteoglycans). SS: non-functionalized human dermal skin substitutes, FSS: functionalized human dermal skin substitutes containing different types of NLCs (AMK, SCM or CTR) at different concentrations (10, 100, 300 or 1000 µg/ml). Scale bar: 10 µm

To determine the effect of functionalization on the biosynthetic activity of the cells immersed in the FSS, synthesis of collagen fibers and proteoglycans was analyzed by picrosirius and alcian blue histochemistry, respectively. Results showed that the amount of these ECM proteins was very low in controls and FSS, with no differences among groups (Fig. 3). First, collagen fibers were mostly absent in all groups, with very few signals restricted to the intracellular space of the stromal cells. However, proteoglycans were comparatively more abundant than collagen fibers, although their expression was very low at 24 and 48 h and was also restricted to the cell cytoplasm. No differences were found between FSS and control samples.

### Antibacterial efficiency of FSS

**Fig. 4:**
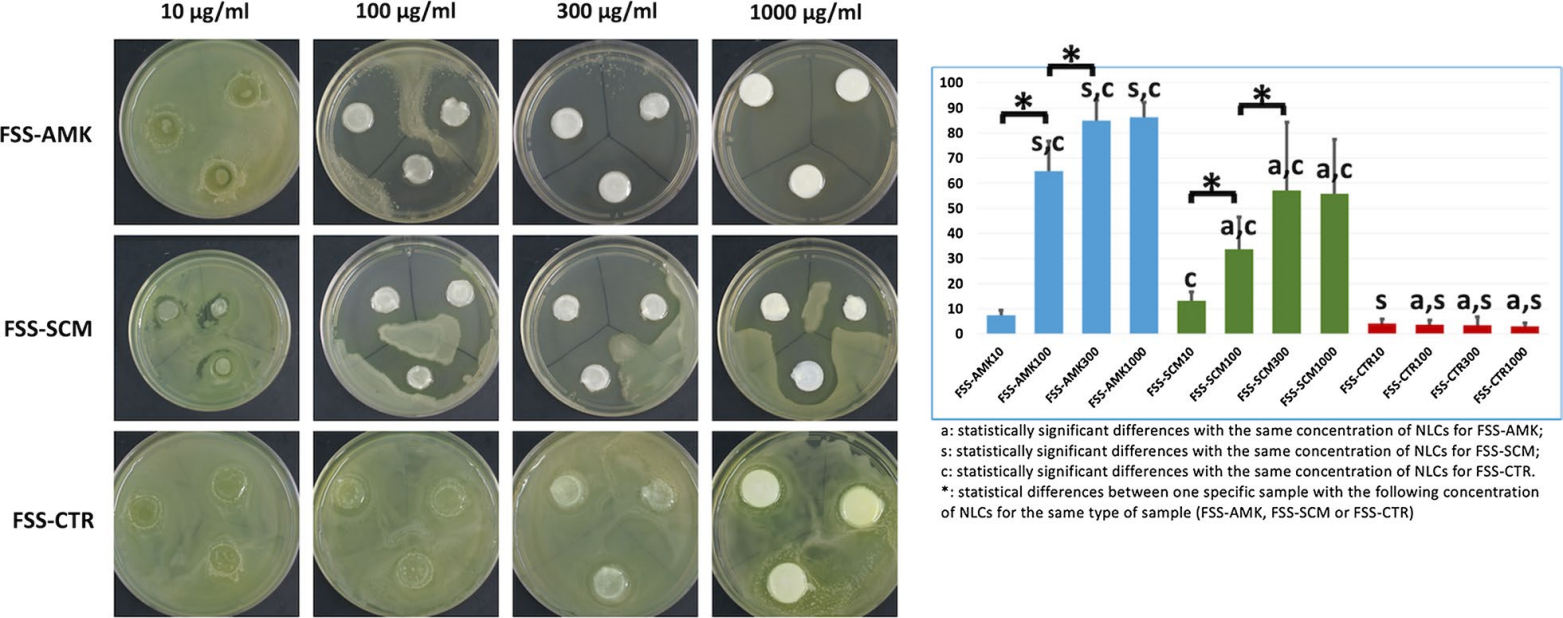
*P. aeruginosa* inhibition area generated by each type of FSS. Representative images are shown in the left panel, and quantified results are shown at the right. For quantification, results are shown as average percentage values with error bars corresponding to standard deviations

To assess the antibacterial effect of functionalized tissues, FSS containing the different types of NLCs were cultured on surfaces seeded with live *P. aeruginosa*. Results showed that the inhibition area (Fig. 4) was highly related to the antibiotic dose, and a statistically significant correlation between concentration and inhibition area was found for FSS-AMK and FSS-SCM samples (p = 0.00013 and 0.00280, respectively), but not for FSS-CTR (p > 0.05), as no antibiotic was loaded in the NLCs embedded within these substitutes. When the type of antibiotic-loaded NLCs was considered, the inhibition area was significantly related to the antibacterial efficiency of FSS (p < 0.00001 for the Kruskal–Wallis test), with significant differences between FSS-AMK and FSS-CTR (p < 0.00001 for the Mann–Whitney test) and between FSS-SCM and FSS-CTR (p < 0.00001 for the same test), with no differences between FSS-AMK and FSS-SCM (p > 0.05). For specific concentrations, the highest inhibitory efficiency corresponded to FSS-AMK300 and FSS-AMK1000, with no significant differences between these two concentrations. Interestingly, the efficiency was higher for each concentration of FSS-AMK as compared to the same concentration of FSS-SCM, except for 10 µg/ml (Fig. 4).

### Biomechanical properties of FSS

Analysis of the biomechanical properties of the different human dermal skin substitutes generated in this work showed that the type and concentration of NLCs could influence these properties.

**Fig. 5:**
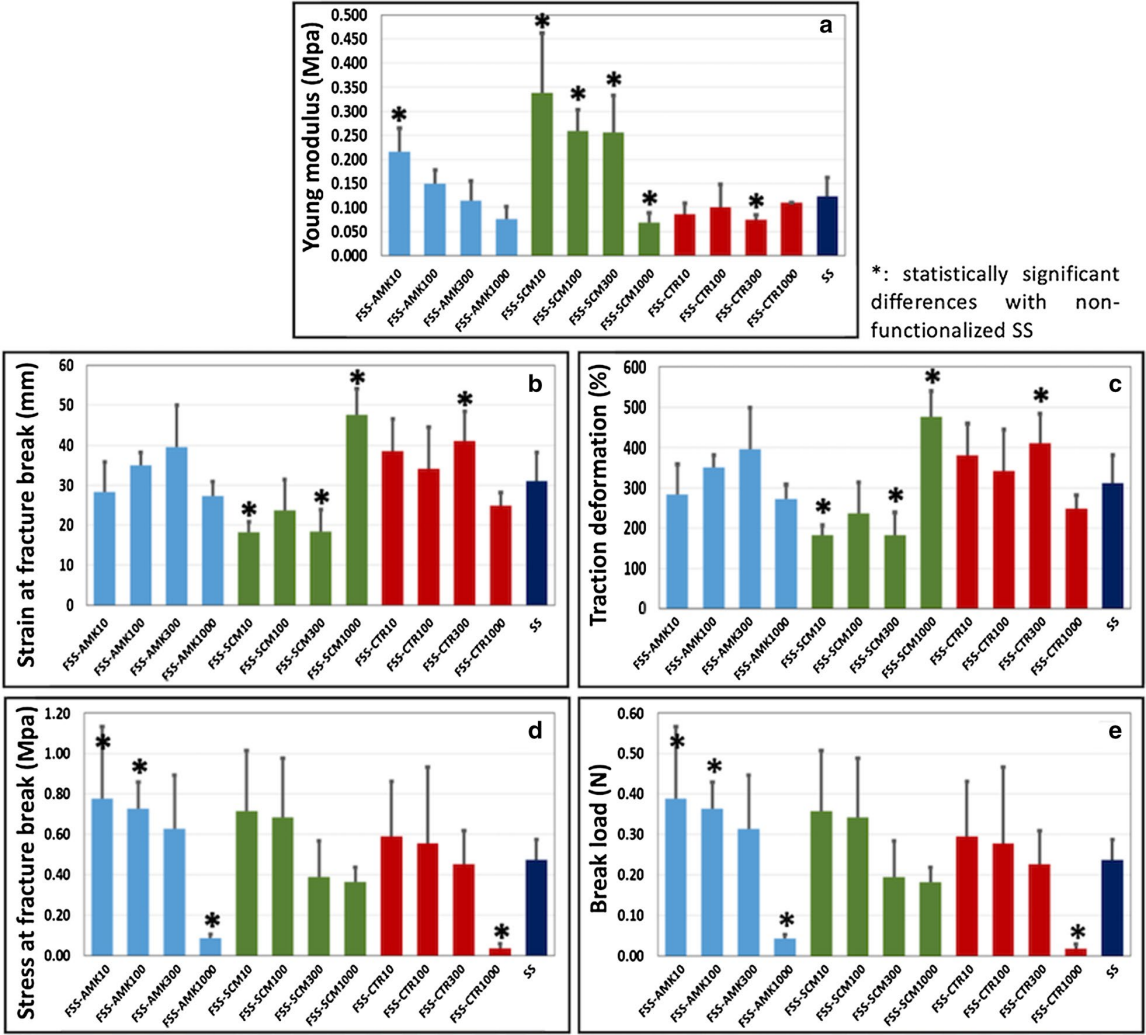
Biomechanical properties of control (SS) and functionalized samples (FSS) generated with different types and concentrations of NLCs. **a**: Young modulus, **b**: strain at fracture break, **c**: traction deformation, **d**: stress at fracture break, **e**: break load. Results are shown as mean values, and error bars correspond to standard deviations

In the first place, the Young modulus of controls and FSS was analyzed (Fig. 5a). Results showed that control non-functionalized SS had a Young modulus of 0.1223 ± 0.0398 MPa. When bioartificial tissues were functionalized with 10 µg/ml of AMK-NLCs and 10, 100 and 300 µg/ml of SCM-NLCs, a significant increase in this parameter was found as compared with SS. In contrast, FSS-SCM1000 and FSS-CTR300 samples showed a significant decrease in this parameter. Non-significant differences with SS were found for the rest of FSS. Interestingly, a significant correlation between the NLC concentration and the Young modulus was found for FSS-AMK samples (p < 0.001), but not for FSS-SCM or FSS-CTR.

In the second place, to determine the deformation capability of each sample to a traction load before rupture, the strain at fracture break and the traction deformation parameters was evaluated (Fig. 5b, c). In this regard, the strain at fracture break of non-functionalized SS was 31.12 ± 6.99 mm, corresponding to 311.1 ± 69.9% of traction deformation. When the dermal substitutes were functionalized, a significant increase of both parameters was shown in FSS-SCM1000 and FSS-CTR300, and a significant decrease in FSS-SCM10 and FSS-SCM300. For FSS-AMK, a non-significant increasing trend up to 300 µg/ml was found. Correlation with the concentration of NLCs was statistically significant for FSS-CTR (p = 0.0306), but not for FSS-AMK or FSS-SCM.

Finally, the stress at fracture break and the break load parameters of controls and FSS was assessed to determine the force that each sample was able to bear before fracturing (Fig. 5d, e). SS showed 0.4738 ± 0.0999 MPa for the stress at fracture break and 0.2369 ± 0.05 N for the break load. Functionalized dermal substitutes showed a significant increase of both parameters in FSS-AMK10 and FSS-AMK100, and a significant decrease in FSS-AMK1000 and FSS-CTR1000. A significant correlation was found between the concentration of NLCs and the stress at fracture break or the break load for the three types of NLCs (AMK-NLCs, p = 0.0055; SCM-NLCs, p = 0.0268; CTR-NLCs, p = 0.0024).

## Discussion

As the largest organ in the body, the skin is the primary protective barrier between an individual and the surrounding environment. Among the main conditions severely affecting the human skin, large burns are life-threatening situations. One of the therapeutic tools showing promising results in these patients is the UGRSKIN model [16], but further research is still in need to improve the efficiency of this ATMP.

Burn injuries typically destroy the skin integrity, and avascularity, protein rich environment and generalized immune suppression make burn wounds particularly susceptible to infections [17–20]. One of the most common bacterial species affecting burn patients is *P. aeruginosa* [17–20], and its presence may significantly worsen prognosis and survival rate. To overcome these limitations, different innovative approaches have been described, such as biomaterial functionalization with graphene oxide [32] or montmorillonite [33]. However, the inability to control severe burn-related infections is still challenging.

In the present study, we generated an improved dermal skin substitute by combining the regenerative properties of the UGRSKIN model with antibiotic-loaded NLCs. These nanoparticles already demonstrated to be an effective drug delivery system suitable for the treatment of *P. aeruginosa* infections [24], with no associated in vivo side effects [26, 27].

Our results first demonstrated that functionalization with NLCs was very effective, and FSS showed strong antibacterial effectiveness on *P. aeruginosa* cultures. In the first place, we found that the antibacterial activity of the FSS was dose-dependent with a strong correlation between the concentration of antibiotics and the bacterial inhibition area. This finding was unsurprising, since dose-dependent antibacterial effect is one of the main characteristics of most known antibiotics [34], although this is the first time that this phenomenon is demonstrated for nanoencapsulated antibiotics combined with fibrin-agarose hydrogels. As previously demonstrated, the use of antibiotic-loaded NLCs is associated to a significant reduction of the antibiotic doses needed for an efficient ex vivo and in vivo effect, and twofold reduction in the inhibition area was obtained as compared to free antibiotics [27].

In the second place, our results showed that both types of antibiotics (AMK and SCM) were very effective against *P. aeruginosa* as compared to controls, and a global comparison between both antibiotics revealed non-significant differences. In general, these results suggest that both, AMK and SCM loaded into NLCs, could be promising tools that may provide antimicrobial properties to functionalized dermal substitutes in order to treat or prevent bacterial wound infections. However, a detailed comparison of specific concentrations showed that AMK was more efficient than SCM for the highest concentrations. Moreover, considering that *P. aeruginosa* is one of the bacteria responsible for antimicrobial resistance (AMR), a major global threat facing public health [35], the fact that our improved dermal substitute can be functionalized with AMK and SCM, two of the few antibiotics effective against gram-negative resistant bacteria, provides added value to this ATMP.

In general, these results confirm the ex vivo usefulness of the functionalized models of human dermis described in the present work, and suggest that both antibiotics could be effective for preventing *P. aeruginosa* infections of the wound bed. The fact that both antibiotics were efficient opens the door to the possibility of using one of them as a first-line treatment for severe skin burns, and reserving the other antibiotic as a second-line option, although a combination of both in a single FSS could also be an option. Future studies should be carried out to determine the most adequate composition of FSS for clinical use.

On the other hand, one of the most important requirements for therapeutic use of biomaterials in tissue engineering is biosafety [36]. In this regard, evaluation of the side effects of functionalization is necessary before FSS can be clinically used. For this reason, we analyzed several parameters directly related to biosafety in FSS, including biocompatibility, cell viability, proliferation and ex vivo function. Our results first showed that most cells in the FSS were alive, with no structural membrane damage demonstrated by quantification of free DNA released to the culture medium and no morphological alterations as determined by HE staining. Cell viability was higher than 90% in all conditions, confirming the high biocompatibility of these nanoparticles [27]. Although cell proliferation and metabolic function, as determined by WST-1, was partially reduced at 24 h, cells were able to recover their initial condition after 48 h, which is in agreement with our immunohistochemical results for KI67. Previous results demonstrated that cells immersed in hydrogels for tissue engineering use could need some time to adapt to the new culture conditions and may require some days to fully recover the initial cell number and function [37, 38]. All these results confirm that FSS fulfill the biocompatibility requirements of bioartificial tissues for future clinical, as previously described [27].

These findings are in agreement with the immunohistochemical analyses of the dermal skin substitutes showing negative reaction for caspase 7, a marker of apoptotic cell death, which reinforces the non-toxic effects of the NLCs used in this work. Similarly, we found a positive immunohistochemical reaction for the cell proliferation markers KI67 and PCNA, confirming the functional status of the cells in all FSS groups. The different behavior found for PCNA and KI67 could be explained by the longer half-life of the PCNA protein (at least 20 h), which implies that nuclei could continue to express PCNA even after completing the cell cycle [39], whereas KI67 is known to be more sensitive and specific [40].

After demonstrating that the nanoparticles used in the present work were safe for the cells immersed in the dermal substitute, we analyzed the influence of these NLCs on the biosynthetic capability of the FSS cells. Results showed that the synthesis of collagen and proteoglycans, the most important extracellular matrix components synthetized by the dermal substitute of a bioartificial skin [15], were actively synthetized by the FSS cells, with no differences with controls. Although longer follow-up studies should be carried out, these preliminary results confirm that functionalization of the human dermal skin substitute did not affect negatively the human dermal cells. Fibroblasts remained viable, morphologically normal and biosynthetically functional as they were in non-functionalized human dermal skin substitutes.

In addition, bioengineered tissues should resemble the native tissues not only at histological level, but also from a biomechanical standpoint. For this reason, in the present work we performed a biomechanical characterization of the FSS in order to determine if functionalization may have modified the main properties of these bioartificial tissues, and to assess if the incorporation of the different types of NLCs could have changed the three-dimensional structure of the biomaterial and, therefore, its biomechanical properties. A proper biomechanical characterization of the novel FSS would contribute to future clinical translation, since a comprehensive characterization of novel tissue substitutes is a requirement of all National Medicines Agencies before clinical use [3, 36, 41, 42]. Thus, once effectivity and safety of the functionalized human dermal skin substitutes were proven, we decided to test the biomechanical properties of the FSS. In general, our results showed that both the type and concentration of NLCs could influence the biomechanical properties of the human dermal skin substitutes.

Interestingly, we found that functionalization was able to modify the Young modulus of the FSS in a dose and type-dependent manner. In this regard, we found a statistically significant increase of this parameter in FSS containing the lowest concentrations of AMK and SCM nanoparticles as compared to the SS group, whereas the highest concentrations showed the opposite effect. Strikingly, the use of CTR-NLCs showed a different behavior. As the Young modulus is directly linked to the biomaterial stiffness [6], these results suggest that antibiotic-loaded NLCs could be used not only for tissue functionalization, but also to improve the biomechanical properties and stiffness of human tissue substitutes. Related to the Young modulus, we analyzed the stress at fracture break and break load of each type of bioengineered human skin. Both parameters are associated to the maximum stress force that the bioartificial tissues can resist before irreversible damage [6]. As for the Young modulus, we found that functionalization with the lowest concentrations of nanoparticles, especially in the case of AMK, were able to improve the stiffness of the FSS, although a high concentration of particles resulted in the opposite effect.

Other critical biomechanical parameters of bioengineered human tissues are the strain at fracture break and the traction deformation. Both parameters are related to the deformation that the bioartificial tissue can sustain before irreversible damage, and are strictly dependent on the tissue elasticity [6]. Remarkably, our results showed that these parameters were also dependent on the type and concentration of NLCs. Whereas low concentrations of nanoparticles were associated to a loss of elasticity, we found a significant increase of both parameters for the highest concentration of SCM-NLCs and in FSS-CTR300, suggesting that the plastic deformation capability of FSS can be modified upon functionalization.

Altogether, our biomechanical results show that functionalization of the dermal skin substitute can significantly tune the biomechanical behavior of this bioartificial tissue. In this line, not only the concentration, but also the type of encapsulated antibiotic, are associated to these biomechanical changes. This new generation of functionalized dermal skin substitutes could improve the biomechanical properties of the current UGRSKIN model by combining an optimum type and concentration of antibiotic-loaded nanoparticles.

The reasons why functionalization is able to increase or decrease the biomechanical properties of FSS requires further research. However, we may hypothesize that NLCs could associate to fibrin-agarose monomers during polymerization of the hydrogel and increase the biomechanical stability of the 3D fiber mesh, thus contributing to hold the incoming forces with higher efficiency. In fact, we previously found that different types of nanoparticles can be adsorbed on the fibrin fibers and increase the attraction between adjacent fibers, resulting in a significant improvement of the biomechanical properties of the hydrogel [43]. On the opposite, it is likely that an excess of particles may interfere the fibrin-agarose polymerization process and alter the mesh polymerization. Regarding differences among the types of nanoparticles, previous reports showed that AMK and SCM have different hydrophobicity [27]. While AMK has lower water solubility and a higher affinity for the lipid phase, SCM typically has higher encapsulation efficacy and slower drug released profile. If this factor may influence linkage of NLCs to the fibrin-agarose fibers should be determined in future studies.

## Conclusions

In conclusion, this ex vivo study demonstrated that fibrin-agarose human dermis substitutes can be functionalized with antibiotic-loaded NLCs, and that FSS show improved bioactive antibacterial properties as compared to controls, with no side effects for the cells embedded in the bioartificial tissue. Furthermore, functionalization was able to improve some of the main biomechanical parameters of FSS. Although future studies are in need to demonstrate the real effect of FSS in vivo, these preliminary promising results support the future clinical use of the novel functionalized dermal skin model.

## Materials and methods

### Antibiotic-loaded nanoparticle preparation

AMK- or SCM-loaded NLCs (AMK-NLCs or SCM-NLCs) and empty nanoparticles used as controls (CTR-NLCs), were prepared by the hot melt homogenization technique following a modified protocol reported by Pastor et al*.* [26], in which the sonication time was increased up to 30 s. Briefly, Precirol^®^ ATO 5 (Gattefossé, Madrid, Spain) and Miglyol 182 N/F (Gattefossé, Madrid, Spain) were melted with the antibiotic (AMK or SCM) to achieve the oily phase (10:1:1). In the case of empty nanoparticles, no antibiotic was used. Meanwhile, the aqueous phase was prepared dissolving Tween^®^ 80 (Panreac Química, Barcelona, Spain) at 1.3% (w/v) and Poloxamer 188 (BASF, Ludwigshafen, Germany) at 0.6% (w/v) and then the solution was tempered above the melting temperature of the solid lipid. Both phases were mixed and emulsified under sonication for 30 s at 50 W. The resulting emulsion was then gradually cooled down and kept at room temperature for 1 h under gentle stirring. Trehalose was added as cryo-preserving agent prior to the freeze-drying step in a final concentration of 15% (w/w) of the weighed lipid. Nanoparticles presented both, encapsulated and non-encapsulated antibiotics (on the nanoparticle surface), as no washing step was included during nanoparticle preparation.

### Antibiotic-loaded nanoparticle characterization

Nanoparticles were characterized in size and dispersion (SPAN) by means of Nanosight NS300 Zetasizer Nano ZS (Malvern Panalytical Ltd, Spain) based on nanoparticle tracking analysis (NTA).

Drug loading was calculated by means of the following formula:

$$ Drug\;loading = \frac{{Initial\;Drug\;Amount\left( {mg} \right)}}{{Total\;Batch\;Weight(mg)}} $$

Besides, encapsulation efficiency was estimated indirectly by quantifying the amount of antibiotic present in the supernatant as already described by Vairo et al. [27]. In short, 5 mL of the cooled suspension were centrifuged in an Amicon^®^ centrifugal filtration unit (100 kDa molecular weight cut off, Millipore) for 15 min at 2500 rpm to analyze the amount of nonencapsulated drug. SCM amount was determined by using the Micro BCA™ Protein Assay Kit (Thermo Fisher Scientific, Spain) following purchaser instructions. AMK was determined by the ultraviolet–visible (UV–vis) spectrophotometric technique for fluorescamine derivatization. Once quantified, encapsulation efficiency was measured according to the following formula:

$$  EE\left( \%  \right) = \left( {\frac{{Initial\;Drug\;Amount - Non\;Encapsulated\;Drug}}{{Initial\;Drug\;Amount}}} \right) \times 100  $$

Samples were analyzed in triplicates to obtain an accurate mean, except for the drug loading.

### Generation of non-functionalized human dermal skin substitutes (SS) by tissue engineering

Primary cell cultures of dermal fibroblast cells were obtained from skin biopsies taken from healthy donors. Biopsies were carefully rinsed in phosphate-buffered saline and enzymatically digested in a 2 mg/ml *Clostridium histolyticum collagenase I* (Gibco–Thermo Fisher Scientific, Waltham, MA) solution at 37 °C for 6 h to obtain primary cell cultures of skin fibroblasts following previously described protocols [14, 15]. Isolated human dermal fibroblasts were then cultured in Dulbecco’s modified Eagle’s medium -DMEM- (Merck Life Science, St. Louis, MO) supplemented with 10% fetal bovine serum (Merck Life Science) and 1% antibiotics/antimycotics (100 U/mL penicillin G, 100 mg/ mL streptomycin and 0.25 mg/mL amphotericin B; Merck Life Science) under standard cell culture conditions.

Bioengineered human dermal skin substitutes (SS) were generated using fibrin-agarose biomaterials as previously described [7, 9, 11, 14, 15, 28]. In brief, the following components were mixed per each ml of dermal skin substitute: 760 µl of human plasma -as a fibrin source-, 75 μl of DMEM containing 140,000 cultured human fibroblasts, 15 µl of tranexamic acid -as antifibrinolytic agent- (Amchafibrin, Fides-Ecofarma, Valencia, Spain), 50 μl of a 2% solution of type VII agarose (Merck Life Science) melted in PBS, and 100 μl of 1% CaCl_2_ solution (Merck Life Science) -to promote fibrin polymerization-. The mixture was aliquoted in culture plates and allowed to jellify for 24 h in a 37 ºC cell incubator. Then, each type of bioartificial tissue [SS and functionalized human dermal skin substitutes (FSS)] was subjected to plastic compression nanostructuration techniques as previously described [6, 11, 14].

### Generation of functionalized human dermal skin substitutes (FSS) by tissue engineering

FSS were generated as described for SS, but a specific amount of each type of nanoparticle (final concentration of 10, 100, 300 and 1000 µg/ml) was incorporated to the hydrogel mixture right before inducing jellification of the solution by adding CaCl_2_ in order to generate the following types of FSS:

- FSS functionalized with AMK-NLCs at a final concentration of 10, 100, 300 and 1000 µg/ml (FSS-AMK10, FSS-AMK100, FSS-AMK300 and FSS-AMK1000, respectively).
- FSS functionalized with SCM-NLCs at a final concentration of 10, 100, 300 and 1000 µg/ml (FSS-SCM10, FSS-SCM100, FSS-SCM300 and FSS-SCM1000, respectively).
- FSS functionalized with CTR-NLCs at a final concentration of 10, 100, 300 and 1000 µg/ml (FSS-CTR10, FSS-CTR100, FSS-CTR300 and FSS-CTR1000, respectively).

FSS were aliquoted and incubated at 37 ºC as performed for SS, and nanostructuration was then applied.

### Ex vivo analysis of biocompatibility of FSS

To determine the ex vivo biocompatibility of the bioengineered dermal skin substitutes, SS and FSS were kept under standard culture conditions for 24 h and 48 h. After each time point, cell death was assessed by quantifying free DNA released from ruptured cells to the culture medium using a NanoDrop 2000 UV–Vis Spectrophotometer (Thermo Fisher Scientific) as previously described [29]. In addition, cell function was analyzed by quantifying the mitochondrial dehydrogenase activity in live cells using the cell proliferation and viability reagent WST-1 (Roche Diagnostics, Mannheim, Germany) [29]. In both cases, live cells grown in basal medium were used as positive controls (CTR +), whilst cells incubated in 1% triton X-100 were used as negative controls (CTR–). Results obtained in the experimental groups were normalized to controls.

### Histological analysis

To evaluate the effects of NLC functionalization on the dermal fibroblasts included in the dermal substitutes, cells were histologically evaluated after 24 and 48 h. SS and FSS were fixed in formalin, dehydrated and embedded in paraffin by following standard histology protocols. All samples were included in a tissue array and 5 µm-sections were obtained with a microtome. Sections were dewaxed, rehydrated and stained with hematoxylin and eosin (HE) to study cell morphology and structure. To assess cell function, viability and proliferation, histochemical and immunohistochemical analyses were carried out using standard methods and techniques.

For immunohistochemistry, dewaxed tissue sections were incubated with specific primary antibodies anti-caspase 7-for apoptotic cell death- (Abcam, ab69540; dilution 1:100), PCNA (Master Diagnostica, Granada, Spain; MAD-000903QD) and KI67 (Master Diagnostica, Granada, Spain; MAD-000310QD) -both, for cell proliferation-. Then, samples were washed and incubated in secondary anti-mouse or anti-rabbit antibodies (Vector Laboratories, MP-7402-0 or MP-7401-50), and the immunohistochemical signal was revealed using a diaminobenzidine (DAB) development kit (Vector Laboratories). Samples were then briefly contrasted with Harris hematoxylin and mounted using glass coverslips. To identify the synthesis of fibrillar and non-fibrillar components of the dermis extracellular matrix by cells immersed in the different dermis substitutes, histochemical methods for collagen fibers (picrosirius red) and proteoglycans (alcian blue) were used as previously described [30, 31].

### Analysis of the anti-microbial activity of FSS

Antimicrobial activity of FSS was determined by culturing each type of dermal substitute on a bacterial biofilm consisting of *Pseudomonas aeruginosa*. First, commercially-available *P. aeruginosa* strains were purchased from Merck Life Science (NCTC 10,662 Lenticule^®^ discs). Strains were precultured in Luria–Bertani (LB) broth at 37ºC until turbidity bacterial suspension was achieved. 100 µl of this bacterial suspension was then inoculated on LB-agar plates and allowed to dry. Then, each human dermal skin substitute (SS and FSS at increasing concentrations) was placed on top of the bacterial LB-agar surface and cultured for 24 h in a cell incubator at 37 ºC. The inhibitory activity of each experimental group was then calculated by measuring the area of bacterial growth inhibition with the ImageJ software. Samples were analyzed in triplicates.

### Biomechanical characterization of dermal skin substitutes

To determine the effect of NLC functionalization on the biomechanical properties of each dermal skin substitutes, SS and FSS were subjected to tensile tests using an electromechanical material testing instrument (Instron, Model 3345-K3327) as previously described [3, 6, 11]. In brief, dermal skin substitutes were sectioned to a rectangular shape and oriented with their length along the direction of the tension. Each experimental group was clamped at each end of the instrument device, leaving a constant distance of 1 cm between the clamps and all tests were run at a constant strain rate of 5 mm/min at room temperature. Young modulus was calculated as the tangent modulus of the initial, linear portion of the stress–strain curve of each experimental run, while the stress at fracture break (σ break) and the strain at fracture break (ε break) values were determined by selecting the point of the stress–strain curve where the fracture occurred. Traction deformation and break load were automatically calculated by the instrument. A 50-N Instron load cell was used to obtain the data for the stress–strain curves. Calculation of the average value and standard deviation (SD) of the results for each experimental run was operated automatically, using the Instron Blue Hill 2 Material Testing software.

### Statistical analysis

Non-parametric statistical tests were used for all comparisons because none of the study variables were normally distributed. To compare several groups of samples within the same variable, we used the Kruskal–Wallis test. Then, post-hoc comparisons between two specific groups of samples were carried out using Mann–Whitney tests. To determine the correlation between two different distributions, we used the Kendal tau correlation test. All statistical comparisons were performed using the RealStatistics software. For all tests, a p value < 0.05 was considered statistically significant for the double-tailed tests.

## Data Availability

Not applicable.

## Abbreviations

NLC: Nanostructured lipid carriers
SCM: Colistimethate
AMK: Amikacin
AEMPS: Agenica Española de Medicamentos y Productos Sanitarios
ATMP: Advanced therapy medicinal product
SS: Human dermal skin substitutes
FSS: Functionalized human dermal skin substitutes
HE: Hematoxylin and eosin
DAB: Diaminobenzidine
LB: Luria–Bertani
SD: Standard deviation
